# *Chlamydia psittaci* pneumonia in Wuxi, China: retrospective analysis of 55 cases and predictors of severe disease

**DOI:** 10.3389/fmed.2023.1150746

**Published:** 2023-08-21

**Authors:** Ying Gao, Yan Wu, Dan Xu, Liang Bao, Xiao Ding, Lei Lv, Chenhui Ma, Tao Bian, Shuguang Han

**Affiliations:** ^1^Department of Respiratory and Critical Care Medicine, Wuxi No.2 People’s Hospital (Jiangnan University Medical Center), Wuxi, China; ^2^Department of Respiratory and Critical Care Medicine, The Wuxi People’s Hospital, Wuxi, China; ^3^Department of Respiratory and Critical Care Medicine, The Xishan People’s Hospital of Wuxi City, Wuxi, China; ^4^Department of Respiratory and Critical Care Medicine, The Wuxi Fifth People’s Hospital (Jiangnan University Medical Center), Wuxi, China

**Keywords:** *Chlamydia psittaci* pneumonia, severe pneumonia, metagenomic next-generation sequencing, retrospective analysis, clinical characteristics, predictors

## Abstract

**Purpose:**

More and more patients with community-acquired pneumonia have been detected with *Chlamydia psittaci* (*C. psittaci*) infected using metagenomic next-generation sequencing (mNGS). Previously, this was unheard of, and several patients presented with severe pneumonia and even required ECMO. We aimed to clarify the clinical characteristics of *C. psittaci* pneumonia and find out if there are any possible predictors of severe *C. psittaci* pneumonia.

**Methods:**

In this retrospective study, we included all confirmed cases of *C. psittaci* pneumonia in Wuxi. Epidemiological, clinical, and radiological features, as well as laboratory data, were collected and analyzed.

**Results:**

We enrolled 55 patients with *C. psittaci* pneumonia, with 30 (54.5%) having a history of exposure to birds or their internal organs. 50 (90.9%) patients were diagnosed by mNGS. Patients with *C. psittaci* pneumonia had many complications, among which, that deserve sufficient attention from clinicians were vascular embolic events (3, 5.5%). High fever was the most common clinical manifestation (41, 74.5%). The majority of patients had a significant increase in neutrophils ratio, neutrophils to lymphocytes ratio (NLR), rapid c-reactive protein, creatine kinase (CK), and lactate dehydrogenase (LDH), as well as a decrease in lymphocytes ratio, albumin, serum sodium, serum potassium, and serum phosphorus. Chest computed tomography scans revealed unilateral pneumonia (70.9%), consolidation (87.3%), air bronchogram (76.4%), and ground-glass opacity (69.1%). The neutrophil ratio, NLR, LDH, and CK were all factors that could identify severe pneumonia. Both AUCs exceeded 0.8; the respective 95% CIs were 0.715–0.944, 0.710–0.963, 0.677–0.937, and 0.718–0.950; all *p* < 0.05 (0.01, 0.001, 0.007, 0.007 respectively). The ORs were 10.057, 9.750, 10.057, and 9.667, respectively; the 95% CIs were 2.643–38.276, 2.339–40.649, and 2.643–38.276, respectively; all *p*-values were less than 0.05 (0.001, 0.002, 0.001, 0.001 respectively).

**Conclusion:**

*C. psittaci* pneumonia is a very complex disease that changes all the time. Some patients showed severe pneumonia. Patients will have a poor prognosis if they are not treated promptly and effectively. We discovered that many clinical indicators were typical. Meanwhile, significant increases in neutrophil ratio, NLR, LDH, and CK predicted severe pneumonia. Timely detection of mNGS provided substantial help for clinical diagnosis and early treatment.

## Background

Humans can be infected by *Chlamydia psittaci* (*C. psittaci*) via the respiratory tract through air or aerosols, resulting in atypical community-acquired pneumonia (CAP) (1). However, *C. psittaci* is challenging to be identified by traditional methods such as microbial culture or microscopic examination. *C. psittaci* pneumonia is often underdiagnosed or misdiagnosed in humans, with an estimated incidence rate of at least 1.03% (2).

*Chlamydia psittaci* infection causes severe pneumonia in some patients (3). The inflammatory reaction imbalance in their body would lead to cytokine storm (4), resulting in diffuse alveolar injury (DAD) and pulmonary capillary endothelial injury, causing acute respiratory distress syndrome (ARDS) (5). Early etiological diagnosis and timely and effective treatment can improve the prognosis of patients. In recent years, metagenomic next-generation sequencing (mNGS) has become increasingly essential for etiological diagnosis. The researchers analyze nucleic acids from bronchoalveolar lavage fluid (BALF), sputum, and other clinical samples, then create a biological reference database and rigorous sequence read mapping and finally identify pathogens like *Mycobacterium tuberculosis* (6–8). Currently, mNGS has gradually been used for the diagnosis of difficult-to-diagnose severe pneumonia (9). Additionally, mNGS can quickly and accurately identify most pathogens, especially atypical pathogens such as Legionella and *C. psittaci*.

With the increasing popularity of mNGS applications, more and more patients with CAP have been detected with *C. psittaci* infected. A multicenter prospective cohort study in China included 329 patients with severe community-acquired pneumonia (SCAP), 264 of whom were immunocompetent, and 8% of whom were infected with *C. psittaci* (10). Previously, this was unheard of. Nowadays, *C. psittaci* pneumonia has become a public health concern in China. According to a comprehensive review of recent papers, it has a variety of clinical features, and the severity of *C. psittaci* pneumonia ranges from mild flu-like symptoms to life-threatening diseases (1, 11). In this article, we attempt to provide an updated description of patients with *C. psittaci* pneumonia. It will deepen the clinicians’ understanding of the diagnosis and treatment of *C. psittaci* pneumonia, diagnose as soon as possible, treat in time, and improve the prognosis.

## Materials and methods

### Enrolled subjects

This retrospective study included 55 patients with *C. psittaci* pneumonia. The inclusion diagnostic criteria for *C. psittaci* pneumonia were as follows: (1) the diagnostic criteria of adult CAP (12); (2) identification of the *C. psittaci* gene fragments through mNGS analysis of the BALF, blood, or sputum, and met the criteria for a positive mNGS result (13); (3) positive results in *C. psittaci*—specific PCR (14, 15); (4) routine microbiological tests, including blood, sputum, and BALF culture were all negative (14, 15). (5) Respiratory failure, defined as a partial oxygen pressure is <60 mmHg or an oxygenation index <300 mm Hg (1 mm Hg = 0.133 kPa).

These patients were admitted from January 1, 2020, to December 31, 2022. These patients were diagnosed and treated at Wuxi Second People’s Hospital, Wuxi People’s Hospital, Xishan People’s Hospital of Wuxi City, and Wuxi Fifth People’s Hospital. Few patients with *C. psittaci* pneumonia in other hospitals in Wuxi; contacted their doctors to learn.

### Procedures

We obtained epidemiological, demographic, clinical, laboratory, management, and outcome data from patients’ medical records. We acquired data from attending doctors, other healthcare providers, and patients, if data was missing from the records or clarification, was required. All data were checked by two physicians (YG and YW).

The *C. psittaci* infection was confirmed by mNGS for 50 BALF samples or positive PCR testing done by the Wuxi Centers for Disease Control and Prevention (CDC) for 5 BALF samples. One of the patients had BALF and blood mNGS testing. The NGS had mainly completed in the following institutions: the Shenzhen Huada Gene Technology Co. Ltd. (Shenzhen, China), Adicon (Hangzhou, China), Darui Diagnostics (Guangzhou, China), and Genskey (Shanghai, China), etc.

Blood, sputum, or endotracheal aspirates were obtained at admission to identify the possible causative bacteria or fungi. In addition, all patients underwent chest computed tomography (CT) and laboratory tests.

### Outcomes

We describe the clinical data, demographics, epidemiological data (such as poultry contact history), primary medical conditions, signs, and symptoms on admission, chest CT findings, laboratory results, comorbidities, mNGS results, treatment received for *C. psittaci* pneumonia, etc. We conducted a statistical analysis of the severe and non-severe groups and discovered the predictive factors of the severe group.

### Statistical analysis

We present continuous measurements as mean (SD) if they were normally distributed, median (IQR) if they were not, and categorical variables as count (%). The corresponding statistical analysis was used separately with a *t*-test, Kruskall–Wallis test, chi-square test, or Fisher’s exact test. In the case of laboratory results, we also assessed whether the measurements were outside the normal range. Risk factors were analyzed using binary logistic regression analysis. The reliability of the predicted value was analyzed using the receiver operating characteristic curve. We used SPSS (version 21.0), RStudio (version 1.4.1106.0), and MSTATA (V 0.5) for all analyses.

## Results

### Patient characteristics

Fifty-five patients with *C. psittaci* pneumonia were included in this study, three of whom were family members, and two others were mother and son.

For the 55 patients, the mean age was 58 (SD13; range 31–87 years) years, and 56.4% of patients were male. More than half of the patients (54.5%) had a documented history of exposure to birds or their internal organs. The majority of patients had chronic diseases. The overall clinical features of the patients are summarized in Table 1.

**Table 1:** Demographics, baseline characteristics, and clinical outcomes of 55 patients with *Chlamydia psittaci* pneumonia.

Patients (*n* = 55)	
*Age (y)*	
Mean (SD)	58 (13)
Range	31–87
*Sex*	
Female	24 (43.6%)
Male	31 (56.4%)
History of exposure to birds or their internal organs	30 (54.5%)
History of exposure to parrot	20 (36.4%)
History of exposure to a pigeon or their internal organs	9 (16.4%)
History of exposure to wild birds	1 (1.8%)
*Chronic medical illness*	
Cardiovascular system diseases	27 (49.1%)
Diabetic	8 (14.5%)
Digestive system disease	2 (3.6%)
Nervous system disease	5 (9%)
History of malignant tumor	3 (5.5%)
Admission to the intensive care unit	3 (5.5%)
*Clinical outcome*	
Discharged	55 (100%)
Died	0 (0)

Data are *n* (%) unless specified otherwise.

On admission, the most prominent symptom was fever, which occurred in almost all patients; most of them had a high fever (41, 74.5%). Other common symptoms were chills and weakness, cough, and expectoration, but the expectoration was only a little yellow-white sputum or white sputum. About 39 (70.9%) patients had wet rales in their lungs. All symptoms and signs are shown in Table 2.

**Table 2:** Clinical characteristics and treatment of 55 patients with *C. psittaci* pneumonia.

Patients (*n* = 55)	
*Signs and symptoms at admission*	
Fever	55 (100%)
High fever (39.1°C–41°C)	41 (74.5%)
Cough	40 (72.7%)
Expectoration	29 (52.7%)
Chills	25 (45%)
Weakness	17 (32%)
Headache	9 (16.4%)
Chest tightness and shortness of breath	7 (12.7%)
Nausea and vomiting	5 (9%)
Loss of appetite	6 (10.9%)
Sore throat	5 (9%)
Rhinorrhoea	4 (7%)
Muscle ache	5 (9%)
Wet rales in the lungs	39 (70.9%)
*Complications*	
ARDS	3 (5.5%)
Acute respiratory failure	16 (29.1%)
Acute renal injury	7 (12.7%)
Acute liver injury	33 (60%)
Septic shock	1 (1.8%)
Gastrointestinal bleeding	2 (3.6%)
Myocardial injury	2 (3.6%)
Atrial fibrillation	3 (5.5%)
Vascular embolic events	3 (5.5%)
Rhabdomyolysis	2 (3.6%)
*Treatment*	
Oxygen therapy	31 (56.4%)
Mechanical ventilation	2 (3.6%)
Non-invasive (i.e., face mask)	2 (3.6%)
Invasive	2 (3.6%)
CRRT	1 (1.8%)
ECMO	1 (1.8%)
Antibiotic treatment	55 (100%)
Moxifloxacin at the beginning of treatment	37 (67%)
Doxycycline at the beginning of treatment	9 (16%)
Nemonoxacin malate at the beginning of treatment	2 (3.6%)
Adjustment to doxycycline	26 (47%)

ARDS, acute respiratory distress syndrome; ECMO, extracorporeal membrane oxygenation; CRRT, continuous renal replacement therapy.

The number of patients with complications was high. The most common was acute liver injury (33, 60%). That deserves sufficient attention from clinicians was vascular embolic events (3, 5.5%). Other complications included ARDS (3, 5.5%), severe lung injury (16, 29.1%), septic shock (1, 1.8%), and others, as shown in Table 2.

### Laboratory findings

On admission, 37 (67.3%) patients had a neutrophil ratio above the normal range. Many patients’ lymphocyte ratios were lower than usual, and the overall neutrophil-to-lymphocyte ratio (NLR) was 8.25 ± 6.9. For the infection index, all patients had rapid c-reactive protein tests, and their median levels were 151.08 ± 70.35 mg/L. An erythrocyte sedimentation (ESR) test was conducted on 47 patients, 46 (97.9%) of whom had levels higher than usual, with a median of 64 ± 29 mm/h. Procalcitonin (PCT) levels were observed to be elevated in 17 (34%) of the 50 patients tested. The characteristics of all inflammatory indicators are listed in Table 3.

**Table 3:** Characteristics of laboratory tests, chest CT, and electronic bronchoscopies of 55 patients with *C. psittaci* pneumonia.

Patients (*n* = 55)	
*Laboratory tests*	
*Blood routine*	
Leucocytes (× 10^9^ per L; normal range 3.5–9.5)	6.64 (2.73)
Increased	7 (12.7%)
Decreased	5 (9%)
Neutrophils ratio (normal range 0.4–0.75)	0.77 (0.14)
Increased	37 (67.3%)
Lymphocytes ratio (normal range 0.2–0.5)	0.16 (0.13)
Decreased	41 (74.5%)
Neutrophil-to-lymphocyte ratio (NLR)	8.25 (6.9)
*Infection-related biomarkers*	
Erythrocyte sedimentation (mm/h; normal range 0.0–15.0)	64 (29)
Increased	46/47 (97.9%)
Rapid c-reactive protein (mg/L; normal range 0.0–10.0)	151.08 (70.35)
Increased	55 (100%)
Procalcitonin (ng/mL; normal range 0.0–0.5)	0.95 (2.26)
Increased	17/50 (34%)
*Blood biochemistry*	
Albumin (g/L; normal range 35.0–55.0)	31.6 (4.8)
Decreased	41 (74.5%)
Alanine aminotransferase (U/L; normal range 8.0–58.0)	56.1 (45.9)
Increased	18 (32.7%)
Aspartate aminotransferase (U/L; normal range 5.0–50.0)	80.6 (112.2)
Increased	31 (56.4%)
Serum creatinine (μmol/L; normal range 35.2–97.5)	71.4 (18.8)
Increased	6 (10.9%)
Blood urea nitrogen (mmol/L; normal range 2.8–7.6)	4.6 (1.7)
Increased	5 (9%)
Creatine kinase (U/L; normal range 26–196)	1258.9 (4980.7)
Increased	35 (63.6%)
Lactate dehydrogenase (U/L; normal range 109–245)	356.9 (218)
Increased	39 (70.9%)
Serum sodium (mmol/L; normal range 136.0–145.0)	132.0 (18.98)
Decreased	30 (54.5%)
Serum potassium (mmol/L; normal range 3.50–5.30)	3.47 (0.43)
Decreased	31 (56.4%)
Serum phosphorus (mmol/L; normal range 0.90–1.34)	0.77 (0.23)
Decreased	36 (65.5%)
Lactic acid (mmol/L; normal range 0.50–2.20)	1.54 (1.35)
Increased	19/23 (82.6%)
*Urine routine*	
Ketone bodies	21 (48.4%)
Metagenomic next-generation sequencing	51 (92.7%)
BALF mNGS	50 (90.9%)
NGS sequence data of *C. psittaci*	9,821 (43618)
Serum mNGS	1 (1.8%)
*Pathogens detected by BALF NGS*	
*Chlamydia psittaci*	50 (100%)
*Klebsiella pneumoniae*	3 (6%)
*Acinetobacter baumannii*	2 (4%)
*Pseudomonas aeruginosa*	2 (4%)
*Escherichia coli*	1 (2%)
*Haemophilus influenzae*	1 (2%)
*Streptococcus pneumoniae*	1 (2%)
*Citrobacter*	1 (2%)
*Whipple dystrophy*	1 (2%)
*Corynebacterium striatum*	1 (2%)
*Mycobacterium tuberculosis complex*	1 (2%)
*Candida tropicalis*	1 (2%)
*Candida albicans*	4 (8%)
*Cryptococcus neoformans*	2 (4%)
*Pneumocystis jirovecii*	1 (2%)
Human gammaherpesvirus 4	7 (14%)
Human alphaherpesvirus 1	3 (6%)
Human betaherpesvirus 5	1 (2%)
Human betaherpesvirus 6B	2 (4%)
Human betaherpesvirus 7	1 (2%)
Torque teno virus 22	2 (4%)
Chest CT findings	55 (100%)
Unilateral pneumonia	39 (70.9%)
Bilateral pneumonia	16 (29.1%)
Multiple lobe infiltration	22 (40%)
Consolidation	48 (87.3%)
The air bronchogram sign	42 (76.4%)
Ground-glass opacity	38 (69.1%)
Pleural effusion	23 (41.8%)
Multiple nodular shadows	7 (12.7%)
Vacuole shadow	5 (9.1%)
Mediastinal lymph node enlargement	7 (12.7%)
Atelectasis	2 (3.6%)
Electronic bronchoscopy	54 (98.2%)
The bronchial mucosa is hyperemia and edema	54 (98.2%)
BALF mNGS	50 (90.9%)
BALF PCR	4 (7.3%)

Data are *n* (%), *n*/*N* (%), mean (*x*), and standard deviation (SD). Increased means over the upper limit of the normal range and decreased means below the lower limit of the normal range.

All patients had myocardial zymograms, and 35 (63.6%) had elevated creatine kinase (CK) levels, while 39 (70.9%) had increased lactate dehydrogenase (LDH), with one patient having abnormal CK levels (34,035 U/L) and LDH levels (1,273 U/L). Electrolyte abnormalities were prevalent in the majority of patients, with hyponatremia in 30 cases (54.5%), hypokalemia in 31 cases (56.4%), and hypophosphatemia in 36 cases (65.5%), one of whom also displayed abnormal hyponatremia (116.4 mmol/L) and hypokalemia (2.51 mmol/L). Forty-one (74.5%) patients had degrees of albumin reduction. Thirty-three patients had varying degrees of liver function abnormalities, with alanine aminotransferase (ALT) or aspartate aminotransferase (AST) above normal levels; no severe impairment occurred. Only 7 (12.7%) patients had elevated blood urea nitrogen (BUN) or serum creatinine, indicating renal function damage. In addition, lactic acid was increased in 19 patients (19/23, 82.6%), and ketone bodies were detected in 21 patients (48.4%). Which is shown in Table 3.

### Chest CT findings

**Figure 1:**
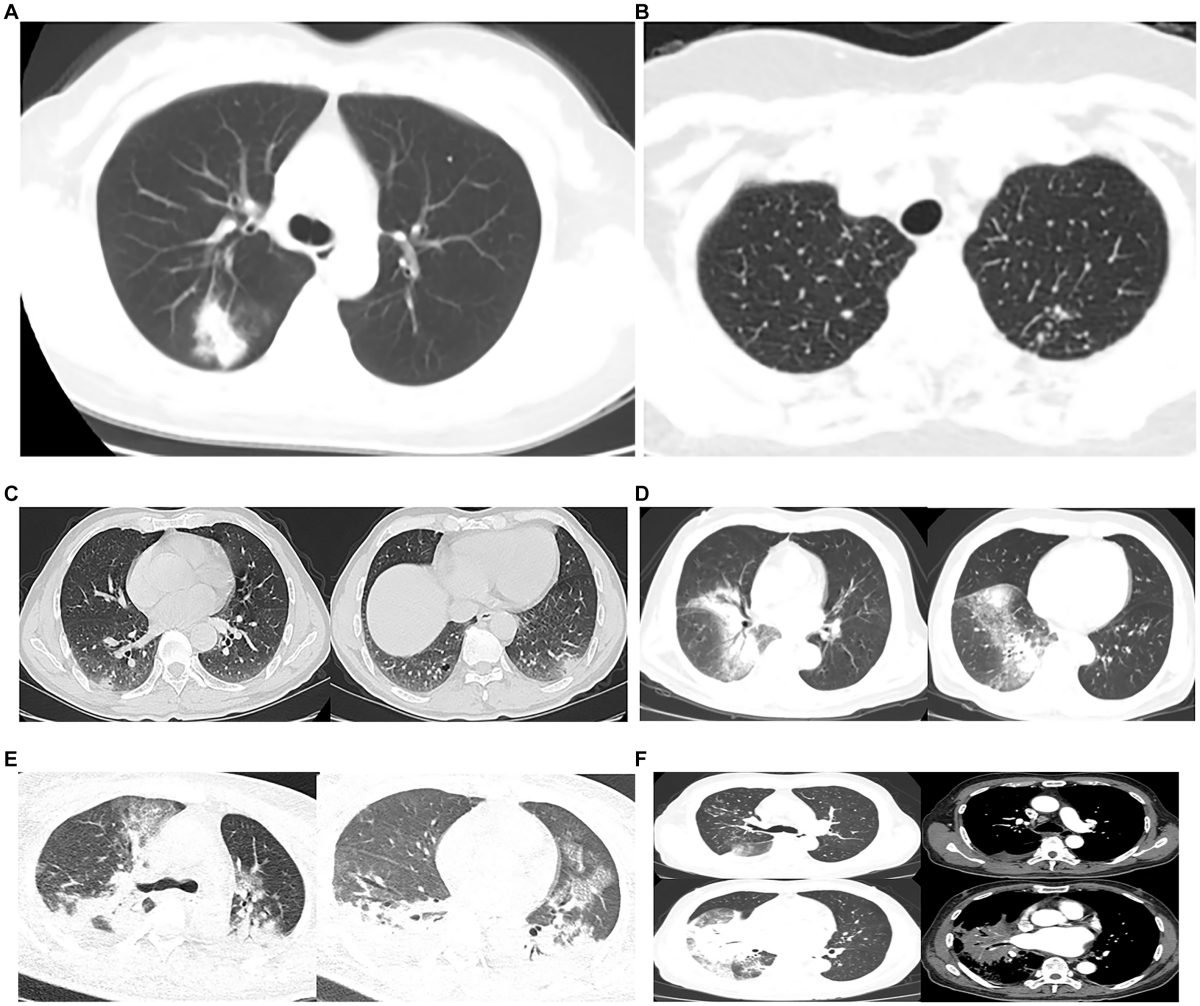
Chest CTs of 6 patients. **(A)**, **(B)** and **(C)** were family members. **(A)** Consolidation and ground-glass opacity was seen in the right upper lung. **(B)** Multiple nodular shadows were seen in both lungs. **(C)** Patchy consolidation and vacuoles in the pleura of both lower lungs, and patchy indistinct shadows in the margin. **(D)** Large patchy consolidation and blurred shadows were seen in the right lung, and an air bronchogram sign was seen inside. **(E)** Severe pneumonia: extensive cloudy flocculent ground glass shadow in both lungs, lamellar consolidation shadow in each lobe of both lungs, air bronchogram sign in them, bilateral pleural effusion, bilateral lower lung atelectasis. **(F)** Severe pneumonia: patchy consolidation shadow could be seen in the right lung, and an air bronchogram sign could be seen inside, cloud flocculent ground glass shadow in the upper and lower lobe of the right lung, enlarged lymph nodes in the mediastinum, and a small amount of effusion in the right chest.

All patients underwent chest CT scans. According to chest CT, 39 (70.9%) patients showed unilateral pneumonia, with just 16 (29.1%) patients showing bilateral pneumonia and 22 (40%) patients showing multiple lobe infiltration. In 48 (87.3%) of the patients, consolidation was observed, and 42 of the patients were observed with air bronchograms as consolidation. The ground-glass opacity was observed in 38 patients (69.1%). Other signs included pleural effusion (23, 41.8%), multiple nodular shadows (7, 12.7%), vacuole shadow (5, 9.1%), mediastinal lymph nodes enlargement (7, 12.7%), and atelectasis (2, 3.6%). All imaging features were shown in Table 3 and Figure 1.

### Etiological results

Among the 55 patients, two of whom were mother and son, and the disease occurred at the same time, the mother did not undergo bronchoscopy, while the other 54 patients underwent bronchoscopy (Table 3). Bronchoscopy revealed congestion and oedema of the bronchial mucosa. A total of 50 patients received mNGS testing of their BALF, and one patient received mNGS testing of both his BALF and blood. The median mNGS sequence number of *C. psittaci* for BALF samples was 9,821 (IQR 4-244,385). The mNGS sequence number in the blood sample was 4. All pathogenic bacteria detected by NGS are listed in Table 3. The BALF from four patients was sent to the Wuxi CDC for PCR testing for *C. psittaci*, and the results were positive. Three of them were family members, the household had two parrots, and the parrots were sick. The living environment was positive for *C. psittaci* based on PCR (Table 3).

Fifty four patients had a sputum culture. Only one patient in the ICU detected Klebsiella pneumonia, and the rest were normal flora or *Candida albicans*. A blood culture was performed on 35 patients, and the results were negative.

### Treatment and outcome

All patients received antibiotic treatment. Before diagnosis, many patients received combination treatment, such as beta-lactam/beta-lactamase inhibitors or cephalosporins coupled with moxifloxacin or doxycycline. When *C. psittaci* was identified as the causative pathogen, doxycycline monotherapy was prescribed to most patients. An allergic response happened in two patients who began therapy with nemonoxacin malate after being adjusted to doxycycline, and the treatment was readjusted to nemonoxacin malate (Table 2).

Three of 55 patients were admitted to the intensive care unit, including two who used mechanical ventilation with an invasive ventilator and one who used a high-flow humidifier. Only one patient was treated with extracorporeal membrane oxygenation. Oxygen therapy was administered to 31 (56.4%) patients in general wards (Table 2).

There was no dead case in our research, which was a benefit. All patients were discharged with normal temperatures, no obvious respiratory symptoms, and normal laboratory results of the re-examination. Following follow-up, 50 patients’ chest CTs showed absorption of pulmonary inflammation, and five patients were informed of improvement *via* telephone.

### Predictors for severe pneumonia

In our study, 16 of 55 cases of *C. psittaci* pneumonia met the criteria for severe pneumonia (12). Patients were divided into two categories, severe and non-severe, and the variables in both groups were statistically analyzed (Table 4). The severe group was found to have more complications than the non-severe group. And the treatment of ventilator or ECMO was only used in the severe group.

**Table 4:** Comparative results of the severe and non-severe groups.

Characteristics	Levels	Severe group (*N* = 16)	Non-severe group (*N* = 39)	*p*
Age (y)	Median (IQR)	62.00 (50.00 to 72.00)	61.00 (48.50 to 66.00)	0.207
Sex	Male	9 (56.2%)	20 (51.3%)	0.970
	Female	7 (43.8%)	19 (48.7%)	—
Peak body temperature (°C)	Median (IQR)	39.95 (38.65 to 40.00)	39.50 (39.05 to 40.00)	0.530
History of exposure to birds or their internal organs	Yes	5 (31.2%)	24 (61.5%)	0.074
	No	4 (25%)	3 (7.7%)	—
Oxygen therapy	Yes	16 (100%)	15 (38.5%)	<0.001
	No	0 (0%)	24 (61.5%)	
Diabetes	Yes	5 (31.2%)	5 (12.8%)	0.134
	No	11 (68.8%)	34 (87.2%)	—
Ketone bodies	Yes	6 (37.5%)	15 (38.5%)	0.065
	No	10 (62.5%)	15 (38.5%)	—
Bilateral lung	Yes	8 (50%)	9 (23.1%)	0.062
	No	8 (50%)	30 (76.9%)	—
Left lung	Yes	4 (25%)	16 (41%)	0.416
	No	12 (75%)	23 (59%)	—
Right lung	Yes	5 (31.2%)	14 (35.9%)	0.986
	No	11 (68.8%)	25 (64.1%)	—
Multiple lobes	Yes	12 (75%)	10 (25.6%)	0.002
	No	4 (25%)	29 (74.4%)	—
*Complications*				
ARDS	Yes	3 (18.75)	0 (0.00)	0.005
Acute respiratory failure	Yes	16 (100.00)	0 (0.00)	<0.001
Septic shock	Yes	1 (6.25)	0 (0.00)	0.115
Hypovolemic shock	Yes	1 (6.25)	0 (0.00)	0.115
Acute renal injury	Yes	5 (31.25)	2 (5.13)	0.008
Acute liver injury	Yes	13 (81.25)	19 (48.72)	0.026
Cardiac insufficiency	Yes	5 (31.25)	0 (0.00)	<0.001
Myocardial injury	Yes	1 (6.25)	1 (2.56)	0.507
Atrial fibrillation	Yes	3 (18.75)	0 (0.00)	0.005
Vascular embolic events	Yes	2 (12.50)	1 (2.56)	0.141
Gastrointestinal bleeding	Yes	2 (12.50)	0 (0.00)	0.024
Rhabdomyolysis	Yes	2 (12.50)	0 (0.00)	0.024
*Laboratory results*				
Leucocytes (× 10^9^ per L; normal range 3.5–9.5)	Median (IQR)	7.01 (5.79 to 8.46)	5.71 (4.74 to 8.11)	0.124
Neutrophils ratio (normal range 0.4–0.75)	Median (IQR)	0.88 (0.83 to 0.92)	0.77 (0.69 to 0.85)	<0.001
Lymphocytes ratio (normal range 0.2–0.5)	Median (IQR)	0.08 (0.06 to 0.12)	0.15 (0.10 to 0.23)	0.001
Neutrophil-to-lymphocyte ratio	Median (IQR)	10.54 (7.35 to 15.92)	4.97 (2.87 to 7.94)	<0.001
Rapid c-reactive protein (mg/L)	Mean ± SD	182.89 ± 62.37	138.03 ± 69.97	0.030
Erythrocyte sedimentation (mm/h)	Mean ± SD	65.42 ± 24.37	63.03 ± 30.70	0.809
Procalcitonin (ng/mL)	Median (IQR)	0.71 (0.19 to 5.37)	0.17 (0.09 to 0.46)	0.008
Albumin (g/L)	Mean ± SD	29.06 ± 3.96	32.68 ± 4.74	0.009
Alanine aminotransferase (U/L)	Median (IQR)	45.15 (33.00 to 81.15)	38.20 (25.00 to 68.60)	0.340
Aspartate aminotransferase (U/L)	Median (IQR)	76.00 (46.65 to 105.90)	43.30 (24.90 to 72.50)	0.023
Lactate dehydrogenase (U/L)	Median (IQR)	427.00 (305.50 to 689.00)	279.00 (203.00 to 326.50)	<0.001
Creatine kinase (U/L)	Median (IQR)	560.00 (246.50 to 1348.00)	87.70 (51.60 to 216.60)	<0.001
Blood urea nitrogen (mmol/L)	Median (IQR)	5.05 (3.42 to 6.71)	4.13 (3.50 to 4.78)	0.101
Serum creatinine (μmol/L)	Mean ± SD	80.49 ± 18.30	67.73 ± 17.95	0.021
Serum sodium (mmol/L)	Median (IQR)	132.45 (129.15 to 137.60)	135.70 (131.90 to 139.15)	0.156
Serum potassium (mmol/L)	Mean ± SD	3.41 ± 0.54	3.52 ± 0.37	0.424
Serum phosphorus (mmol/L)	Mean ± SD	0.74 ± 0.23	0.78 ± 0.24	0.553
Lactic acid (mmol/L)	Median (IQR)	2.37 (1.40 to 2.88)	2.50 (1.70 to 2.60)	0.984

The variables in the table were represented by mean ± SD and the *p*-value was a *t*-test. This variable was described by media (IQR), and the *p*-value was determined using the Kruskall–Wallis test. ARDS, acute respiratory distress syndrome.

The majority of the severe group had bilateral lung inflammation (50% vs. 23.1%, *p* = 0.062) or multiple lobes infiltration (75% vs. 25.6%, *p* = 0.002), but the difference in bilateral lung inflammation was not statistically significant. The neutrophil ratio (0.88 vs. 0.77, *p* < 0.001), NLR (10.54 vs. 4.97, *p* < 0.001), rapid c-reactive protein (182.89 vs. 138.03, *p* = 0.030), PCT (0.71 vs. 0.17, *p* = 0.008), AST (76.00 vs. 43.30, *p* = 0.023), LDH (427.00 vs. 279.00, *p* < 0.001), CK (560.00 vs. 87.70, *p* < 0.001), and serum creatinine (80.49 vs. 67.73, *p* = 0.021) were significantly higher in the severe group than in the non-severe group, and the differences were statistically significant. The lymphocyte ratio and serum albumin level were lower in the severe group than in the non-severe group, and the differences were also statistically significant (0.08 vs. 0.15, *p* = 0.001; 29.06 vs. 32.68, *p* = 0.009). Which is shown in Table 4.

**Figure 2:**
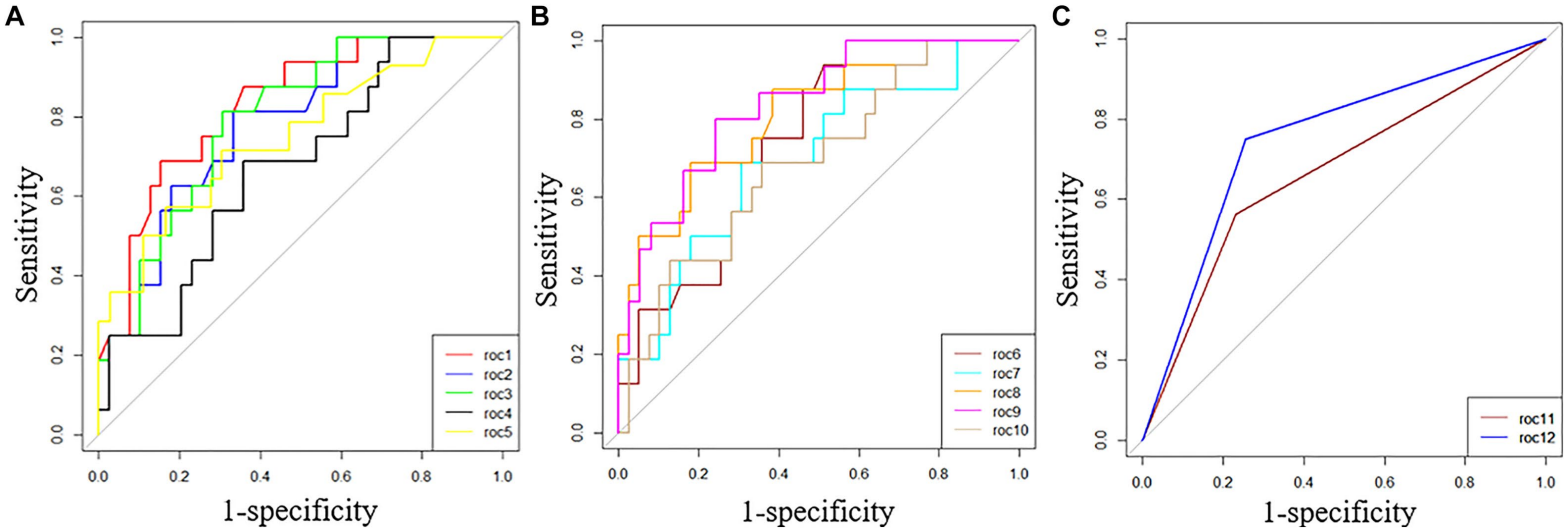
ROC curves of 12 variables predicted for the severe group. **(A)** roc1 = neutrophil ratio; roc2 = lymphocyte ratio; roc3 = NLR; roc4 = rapid c-reactive protein; roc5 = procalcitonin. **(B)** roc6 = serum albumin; roc7 = AST; roc8 = LDH; roc9 = CK; roc10 = serum creatinine. **(C)** roc11 = bilateral lung inflammation; roc12 = multiple lobes infiltration.

**Figure 3:**
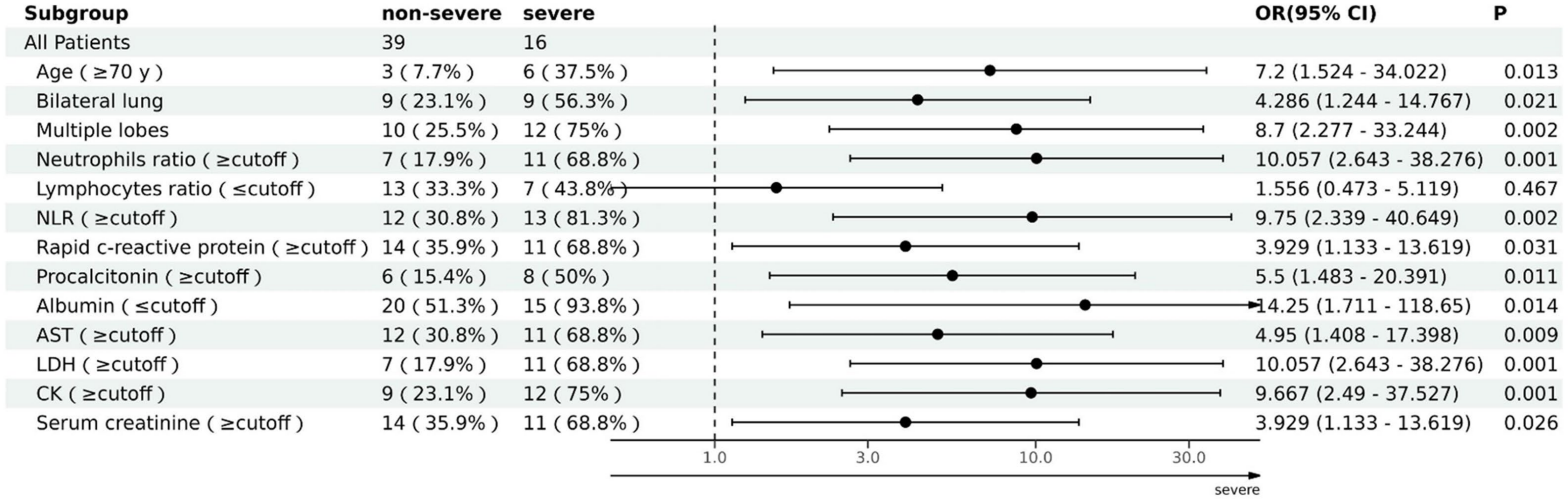
Forest map of 13 risk factors for the severe group.

The ability of these 12 variables to predict the severe group was assessed using the receiver operating characteristic curve (ROC curve) (Table 5 and Figure 2). The ROC curve was employed to determine the cutoff values for each continuous variable, and the cutoff values were utilized to transform the variables into dichotomous variables. Besides, according to ROC curves, the age threshold for both groups was 70 years, which also transformed into a dichotomous variable, but its AUC was 0.650 and *p* > 0.05. The area under the ROC curve (AUC) of neutrophil ratio, NLR, LDH, and CK all surpassed 0.8 (0.855, 0.837, 0.807, and 0.834, respectively); 95% confidence intervals (CIs) were 0.715–0.944, 0.710–0.963, 0.677–0.937, and 0.718–0.950; all *p*-values were lower than 0.05 (0.01, 0.001, 0.007, 0.007 respectively). After that, binary logistic regression analysis showed that all 13 variables (including age) were risk factors for the severe group, but the lymphocyte ratio was not statistically significant (*p* = 0.467), while the others were statistically significant (*p* < 0.05) (Table 5). And the results were presented by the forest plot (Figure 3). Except for the lymphocyte ratio, the forest plot shows that the number of people in the severe group whose variables exceed the cutoff level is considerably higher than that in the non-severe group.

**Table 5:** ROC curve and binary logistic regression analysis results.

Characteristics	ROC curve					Binary logistic regression analysis		
	Cutoff	Youden’s index	AUC	95% CI	*p*	OR	95% CI	*p*
Age (y)	69.5	0.42	0.650	0.441–0.859	0.144	7.2	1.524–34.022	0.013
Neutrophils ratio	0.866	0.521	0.855	0.723–0.987	0.001	10.057	2.643–38.276	0.001
Lymphocytes ratio	0.117	0.479	0.78	0.654–0.907	0.001	1.556	0.473–5.119	0.467
NLR	7.04	0.479	0.837	0.710–0.963	0.001	9.750	2.339–40.649	0.002
Rapid c-reactive protein (mg/L)	155.35	0.299	0.710	0.520–0.900	0.041	3.929	1.133–13.619	0.031
Procalcitonin (ng/mL)	0.635	0.333	0.749	0.589–0.909	0.096	5.5	1.483–20.391	0.011
Albumin (g/L)	33.65	0.425	0.732	0.595–0.870	0.007	14.250	1.711–118.64	0.014
AST (U/L)	64.5	0.380	0.697	0.542–0.852	0.023	4.950	1.408–17.398	0.009
LDH (U/L)	364	0.493	0.807	0.677–0.937	0.007	10.057	2.643–38.276	0.001
CK (U/L)	223	0.513	0.834	0.718–0.950	0.007	9.667	2.490–37.527	0.001
Serum creatinine (μmol/L)	73.75	0.33	0.689	0.535–0.843	0.136	3.929	1.133–13.619	0.026
Bilateral lung	—	0.332	0.666	0.524–0.808	0.055	4.286	1.244–14.767	0.021
Multiple lobes	—	0.494	0.747	0.617–0.876	0.004	8.7	2.277–33.244	0.002

ROC curve, receiver operating characteristic curve; AUC, the area under the ROC curve; CI, confidence interval; OR, odds ratio; NLR, neutrophil-to-lymphocyte ratio; AST, aspartate aminotransferase; LDH, lactate dehydrogenase; CK, creatine kinase.

The factors that could identify severe pneumonia and with good identification ability were found by binary Logistic regression analysis and ROC curves. Those factors were neutrophil ratio, NLR, LDH, and CK, with respective ORs of 10.057, 9.750, 10.057, and 9.667; 95% CIs of 2.643–38.276, 2.339–40.649, 2.643–38.276, 2.490–37.527; and all were statistically significant (all *p* < 0.05; 0.001, 0.002, 0.001, 0.001 respectively).

## Discussion

This is an extended retrospective study of the epidemiology and clinical characteristics of *C. psittaci* pneumonia. A total of 55 patients were examined at four major medical facilities in Wuxi. It presents the latest status of *C. psittaci* pneumonia infection in Wuxi, China. In this paper, we summarized the clinical characteristics of *C. psittaci* pneumonia, identified predictors of severe pneumonia, and pointed out that mNGS is a means to provide early diagnosis.

As everyone knows, *C. psittaci* pneumonia is rare, accounting for one to 8% of CAP cases (2, 16). However, with the increased use of mNGS in recent years, it has been discovered that the detection rate of *C. psittaci* in CAP patients is not low. We can find 14 cases with severe *C. psittaci* pneumonia diagnosed by mNGS (3) and 32 other patients diagnosed with *C. psittaci* pneumonia by mNGS during the COVID-19 pandemic (15). We have reason to suggest that physicians should be vigilant about atypical pneumonia caused by uncommon pathogens such as *C. psittaci*.

The clinical features of these patients in this retrospective study were not the same, but they had many symptoms in common. It was found that the most prominent symptom was fever, which occurred in all patients; most were high fever, their body temperature was over 39.1°C, and most with chills. Other typical symptoms of *C. psittaci* pneumonia include cough, expectoration, weakness, etc. This report is similar to previous ones (17). On the other hand, it differs from previous studies in that most patients had flu-like symptoms, such as fever, fatigue, muscle aches, headaches, and sore throats (7, 18). Aside from fever, other flu-like symptoms were uncommon in this study. The study found that only 12.7% of patients suffered from chest tightness and shortness of breath. The proportion was significantly lower than in other studies (1, 7). Different diagnostic classifications of dyspnea and treatment approaches may explain this.

When a patient with a high fever has a chest CT that shows patchy consolidation with an air bronchogram and ground-glass opacity, *C. psittaci* pneumonia is highly suspected. According to this study, 76.4% of patients had patchy consolidation with the air bronchogram sign and ground-glass opacity on chest CT, the consolidation performance reached 87.3%, and pleural effusion was not uncommon. Several imaging characteristics, such as consolidation of the chest with a bronchogram obtained *via* chest CT, have been reported (19). Most patients have bilateral, middle-lower lobe, or multilobar lesions, usually presenting as consolidation, and have pleural effusions (7). Moreover, bilateral lung consolidation and numerous lobe infiltration were observed primarily in severe cases. However, these signs differ from our understanding of atypical pathogen pneumonia, such as *Chlamydia pneumoniae*. In contrast to *C. psittaci* pneumonia, thickening of the bronchial walls is most common in *M. pneumoniae* pneumonia, followed by nodules (tree-in-bud and centrilobular) (81%) (20). Identifying these CT signs can help diagnose *C. psittaci* pneumonia.

The erythrocyte sedimentation rate, rapid C-reactive protein, CK, and LDH were significantly elevated in laboratory tests. It is known that CK is distributed in many tissues, mainly muscle tissue. High CK levels occur in *C. psittaci* pneumonia, but the mechanism is unclear, and high CK levels are an independent risk factor for severe *C. psittaci* pneumonia (11). Other studies have found that CK levels greater than 174 U/L are a risk factor for severe *C. psittaci* pneumonia (7). Our study found most patients had CK levels higher than 196 U/L, and one of the 55 patients had a significant increase, as high as 34,035 U/L, coupled with rhabdomyolysis in our study. We also found that LDH was significantly increased in most patients (70.9%). This discovery has also been proposed previously (7). The LDH level in the severe group was higher than in the non-severe group, and the difference was statistically significant. These are similar to previous studies (1). Additionally, we found from binomial logistic regression analysis and ROC curve analysis that significant increases in CK and LDH levels may indicate the severity of the disease and both may be predictors of severe pneumonia. This is a discovery that needs to be studied further in the future.

More than half of the patients in this study had electrolyte disorders, primarily manifesting as hypokalemia (54.5%), hyponatremia (56.4%), and hypophosphatemia (65.5%). Since there are few studies on electrolyte disorders, we are interested in learning whether more case reports can provide diagnostic information. Additionally, 21 patients (48.4%) were in ketosis, 5 of whom were diabetic.

It is consistent with the previous reports that the leukocyte increase is not noticeable (4), the neutrophil ratio and NLR increase, the lymphocyte ratio decreases, the PCT increases, the transaminase increases, the creatinine and urea nitrogen increases, and the hypoproteinemia increases, etc. (1, 7). However, in our study, further binomial logistic regression analysis and ROC curve analysis showed that both the increase of neutrophil ratio and NLR were risk factors of the severe group, with ORs of 10.057 and 9.750 respectively, both statistically significant, and AUCs of both were over 0.8, both also statistically significant. This result implies that neutrophil ratio and NLR elevation are indicators of severity and predictors of severe pneumonia.

When a CAP patient with a high fever has the above characteristics, such as a CT showing consolidation with an air bronchogram sign and ground-glass opacity, laboratory tests reveal a significant increase in rapid c-reactive protein, no significant increase in white blood cells, significant increases in CK and LDH, hypokalemia, hyponatremia, hypophosphatemia, and so on. In this patient, there is a high probability of *C. psittaci* pneumonia, and the history of bird exposure should be verified, although this history of bird exposure should be clarified at the initial visit. However, most patients dispute that we should follow up on their history during diagnosis and therapy and are encouraged to recall previous contact information. A study conducted in this study found that 30 patients had a history of exposure to pigeons or parrots or their internal organs. The research discovered that nearly all patients had had close contact with birds or poultry (16). Several reports showed that most patients had a history of poultry contact (7, 15). Among 116 patients studied, only 18 (15.5%) had a history of exposure (1). Sometimes it is difficult to determine the exposure history. As in our study, one patient denied any history of bird contact. However, after being diagnosed with *C. psittaci* Pneumonia, the patient replied that he walked in a park where pigeons were around. This may be an indirect exposure. It reminds us to ask about the patient’s hobbies and habits.

How is the diagnosis of *C. psittaci* pneumonia confirmed when clinical features and exposure history are highly suggestive? We know that conventional methods of diagnosing *C. psittaci* have poor sensitivity and specificity (21). To identify pathogenic bacteria, PCR or mNGS is needed. Nucleic acid PCR assays, which do not rely on culture techniques, are rapid and accurate, but these methods require *a priori* knowledge or assumptions about the pathogen type. mNGS has the main advantage of unbiased sampling, the ability to detect all potential pathogens in the same sample simultaneously and the avoidance of pre-defined detection ranges. Therefore, mNGS has a clear advantage in the diagnosis of unexplained and co-infected lower respiratory tract infections (22). For mNGS testing, BALF is usually used, but sputum or serum can also be tested if bronchoscopy is not an option. In this study, a patient admitted to the intensive care unit was too ill to undergo bronchoscopy in the early stages. Therefore, a serum mNGS test was performed, which revealed a *C. psittaci* infection. Following this test, the appropriate antibiotics were immediately adjusted, enabling the patient to survive and receive effective antimicrobial treatment. Additionally, the symptoms of *C. psittaci* pneumonia are not unique and can be seen in other diseases as well. Furthermore, not all patients can give a history of their exposure. As a result, the diagnosis is not straightforward. In this situation, mNGS is still required. Since mNGS is unbiased and hypothesis-free, it has been widely used in the diagnosis of respiratory tract infections (22). In the case of unexplained pneumonia, mNGS could help identify rare pathogens and shorten diagnosis time. mNGS assays in the lower respiratory tract or pleural effusion samples can provide a rapid and early diagnosis of severe *M. pneumoniae* pneumonia with ARDS (23). In contrast to traditional microbial culture methods, mNGS detects pathogens within 3 days (24). More evidence suggests that mNGS is an effective tool for diagnosing *C. psittaci* pneumonia (7, 25). But for some patients, mNGS testing is unaffordable due to its high cost.

Our study was fortunate to have no deaths among the patients. An intensive care unit admitted three patients, one of whom required tracheal intubation with mechanical ventilation and ECMO, and the outcome was positive. This could be due to the fast etiological diagnosis of these patients using mNGS, which allowed them to receive effective antibacterial therapy. The treatment of *C. psittaci* is straightforward. *C. psittaci* can be treated with tetracyclines, macrolides, and quinolones (26). In this study, each patient received either doxycycline, moxifloxacin, or nenofloxacin malate. A few patients with poor results on oral moxifloxacin were switched to oral doxycycline, and some patients with allergic reactions to oral doxycycline were switched to quinolones. Tetracycline-resistant chlamydia strains have been reported in recent years due to the widespread use of tetracycline in poultry (27, 28). A new generation of quinolones, moxifloxacin is highly effective against *C. psittaci* (1). However, the antibiotics must be personalized to the particular condition.

A total of three patients in our study had vascular embolism events, namely pulmonary embolism, cerebral artery thrombosis, and bilateral lower limb vein thrombosis. The literature search did not yield any relevant articles. It is unclear whether *C. psittaci* infections cause vascular emboli, and further observation and research are needed.

There are several limitations to this study. First, only 55 patients with confirmed *C. psittaci* pneumonia were included; those with strong clinical suspicion but no evidence of pathogens were excluded. As a consequence, some information may be missed. Second, most patients were diagnosed with BALF mNGS but did not receive standard laboratory tests. Not all patients can afford mNGS. Third, several factors regarding the use of medication, such as drug combinations, different types and manufacturers, and irregular application of antibiotics against *C. psittaci*, such as one patient being administered moxifloxacin and doxycycline consecutively, were included. These might lead to bias in the analysis of drug efficacy. Research on these drugs’ efficacy in *C. psittaci* pneumonia requires more in-depth prospective studies.

## Conclusion

There were 55 hospitalized patients with confirmed *C. psittaci* pneumonia in this multi-centre case series in Wuxi, China. We found that many clinical indicators were typical. Meanwhile, significant increases in neutrophil ratio, NLR, LDH, and CK predicted severe pneumonia. Timely detection of mNGS provides substantial help for clinical diagnosis and early treatment. This provides clinicians with a better understanding of the disease and some useful information for the CDC to help them carry out effective epidemiological control measures.

## Data availability statement

The original contributions presented in the study are included in the article/supplementary material, further inquiries can be directed to the corresponding authors.

## Ethics statement

Written informed consent was obtained from the individual(s), and minor(s)’ legal guardian/next of kin, for the publication of any potentially identifiable images or data included in this article.

## Author contributions

TB and SH: conception and design of the experiments. YG, DX, LB, XD, and CM: collection of clinical data. YG and DX: analysis of the data. YG and YW: writing the text of the main manuscript. LB and LL: preparation of tables and figures. All authors contributed to the article and approved the submitted version.

## Acknowledgments

The authors thank all patients involved in the study.

## Conflict of interest

The authors declare that the research was conducted in the absence of any commercial or financial relationships that could be construed as a potential conflict of interest.

## Publisher’s note

All claims expressed in this article are solely those of the authors and do not necessarily represent those of their affiliated organizations, or those of the publisher, the editors and the reviewers. Any product that may be evaluated in this article, or claim that may be made by its manufacturer, is not guaranteed or endorsed by the publisher.
